# Histone trimethylation at H3K4, H3K9 and H4K20 correlates with patient survival and tumor recurrence in early-stage colon cancer

**DOI:** 10.1186/1471-2407-14-531

**Published:** 2014-07-22

**Authors:** Anne Benard, Inès J Goossens-Beumer, Anneke Q van Hoesel, Wouter de Graaf, Hamed Horati, Hein Putter, Eliane CM Zeestraten, Cornelis JH van de Velde, Peter JK Kuppen

**Affiliations:** 1 Department of Surgery, K6-R, Leiden University Medical Center, P.O. Box 9600, 2300 RC Leiden, The Netherlands; 2 Department of Medical Statistics, Leiden University Medical Center, Leiden, The Netherlands

**Keywords:** Histone modifications, Trimethylation, Epigenetics, Colon cancer, Prognosis, Patient survival, Tumor recurrence

## Abstract

**Background:**

Post-translational modification of histone tails by methylation plays an important role in tumorigenesis. In this study, we investigated the nuclear expression of H3K4me3, H3K9me3 and H4K20me3 in early-stage colon cancer in relation to clinical outcome.

**Methods:**

Tumor tissue cores of 254 TNM stage I-III colorectal cancer patients were immunohistochemically stained for H3K4me3, H3K9me3 and H4K20me3 and scored using the semi-automated Ariol system. Cox proportional hazard trend analyses were performed to assess the prognostic value of the combined markers with respect to patient survival and tumor recurrence.

**Results:**

The histone methylation markers only showed prognostic value in early-stage (TNM stage I and II) colon cancer. Therefore, only this patient set (n = 121) was used for further statistical analyses. Low nuclear expression of H3K4me3, and high expression of H3K9me3 and H4K20me3 were associated with good prognosis. In combined marker analyses, the patient group showing most favorable expression (low H3K4me3, high H3K9me3 and high H4K20me3) was associated with the best prognosis. Multivariate trend analyses showed significantly increased hazard ratios (HR) for each additional marker showing unfavorable expression, as compared to the “all favorable” reference group. The HR for disease-free survival was 3.81 (1.72-8.45; p = 0.001), for locoregional recurrence-free survival 2.86 (1.59-5.13; p < 0.001) and for distant recurrence-free survival 2.94 (1.66-5.22; p < 0.001).

**Conclusions:**

Combined nuclear expression of histone modifications H3K4me3, H3K9me3 and H4K20me3 is prognostic in early-stage colon cancer. The combination of expression of the three histone modifications provides better stratification of patient groups as compared to the individual markers and provides a good risk assessment for each patient group.

## Background

In tumor cells, numerous changes in epigenetic regulation of gene expression have been reported [1]. As epigenetic mechanisms are potentially reversible, they represent suitable targets for the development of new anti-cancer therapies. Both DNA methylation and histone modifications might therefore present as possible new biomarkers in cancer. In this study, we investigated the clinical prognostic value of several histone modifications in early-stage (TNM stage I and II) colon cancer.

Epigenetic regulation of gene expression through post-translational modification of histone proteins by methylation plays an important role in many biological processes, including cell-cycle regulation, DNA damage- and stress response, embryonic development and cellular differentiation [2]. The most extensively studied histone methylation sites include histone H3 lysine 4 (H3K4), H3K9 and H4K20. Altered expression of these - and other - histone modifications has been reported in cancer [3]. For example, expression of H3K4me3 was shown to have prognostic value in hepatocellular carcinoma [4] and renal cell carcinoma [5]. Cancer-associated upregulation of H3K9me3 was prognostic in acute myeloid leukemia [6], salivary carcinoma [7] and bladder cancer [8]. Expression of H4K20me3 was shown to be correlated to tumor progression and prognosis in non-small cell lung cancer [9]. Marión *et al*. showed that loss of H4K20me3 contributed to telomere reprogramming and hence a higher tumorigenic potential [10]. As these three histone methylation markers have been found to contribute to the tumorigenic process in various cancers, we hypothesized that these histone modifications would correlate to clinical outcome in colon cancer.

In addition to the individual functions of the histone modifications, they work together regulating gene expression and chromatin structure in different regions of the genome. H3K4me3 and H3K9me3 both regulate gene promoter activity and are mutually exclusive at promoter regions [11]. H4K20me3 and H3K9me3 are both present on pericentric regions [12,13] and are critical for condensation of chromatin at these regions. Both H3K9me3 and H4K20me3 have also been found to be enriched on imprinted genes [14]. The study by McEwen *et al.* also showed that all three histone methylation marks H3K4me3, H3K9me3 and H4K20me3 form a tri-mark signature on imprinting control regions [14]. Based on the overlapping functions of the three histone methylation marks, we hypothesized that combining these three modifications in survival analyses would be more informative than the individual markers with respect to patient survival and tumor recurrence. A combination of high expression of activating histone modification H3K4me3 and low expression of silencing modifications H3K9me3 and H4K20me3 was expected to correlate with poor clinical outcome in colon cancer. Using immunohistochemistry and semi-automated scoring, nuclear expression of H3K4me3, H3K9me3 and H4K20me3 was determined on a tissue microarray of colorectal cancer patients, and subsequently correlated to clinical outcome.

## Methods

### Patient selection

Tumor tissues were collected from a consecutive series of 409 colorectal cancer patients who underwent surgical resection of a primary colorectal tumor at the Leiden University Medical Center (LUMC) between 1991 and 2001. Patients were excluded from the study analyses when patients had a history of cancer other than basal cell carcinoma or *in situ* tumors, had multifocal tumors or received preoperative treatment. Data were right-censored when patients were alive or free of recurrence at their last follow-up date. Patient records information was anonymized and de-identified prior to analysis according to national ethical guidelines (“Code for Proper Secondary Use of Human Tissue”, Dutch Federation of Medical Scientific Societies), and approved by the Medical Ethical Committee of the Leiden University Medical Center (LUMC). In the study cohort, we only included patients with TNM stage I-III tumors (n = 259). Of 254 patients, complete data on all the studied markers were available. This study was performed according to the REMARK guidelines (NCI-EORTC) [15].

### Tissue microarray construction and immunohistochemistry

Formalin-fixed paraffin-embedded (FFPE) tumor tissues from 409 colorectal cancer patients were collected from the LUMC pathology archives and used to construct a tissue microarray (TMA), as described previously [16]. Three tumor tissue cores, and if available one normal tissue core, were included in the TMA for each patient. Sections of 4 μm were cut from each TMA block and used for immunohistochemical (IHC) staining. Histologically normal colon tissues, as determined by an experienced pathologist, from 29 patients were also included and IHC stained. The following antibodies were used for IHC: anti-H3K4me3 (ab8580, ABcam, Cambridge, UK), anti-H3K9me3 (07-442, Millipore, Billerica, MA, USA) and anti-H4K20me3 (ab9053, Abcam). All primary antibodies were used at predetermined optimal dilutions and IHC was performed using a standard IHC protocol [17]. Briefly, endogenous peroxidase was blocked by incubating the sections in a 0.3% solution of hydrogen peroxide (in PBS) for 20 min. Antigen retrieval was performed by heating the sections for 10 min at 95°C in a citrate buffer (pH 6; pH Low Target Retrieval Solution, Dako, Glostrup, Denmark), followed by overnight (16 hrs) incubation of the respective primary antibodies. Staining was visualized using the Dako REAL™ EnVision™ Detection System, Peroxidase/DAB+, Rabbit (Dako). The stained TMA slides were scanned using a 20x magnification on the Ariol system (Leica Microsystems, Wetzlar, Germany), followed by marking the tumor cell areas or normal colon epithelium for each tissue punch upon visual inspection on the computer screen. The Ariol system is specifically designed to recognize cells, nuclei, cell membranes and pixel intensity. For each type of staining (membranous, cytoplasmatic or nuclear), different software packages are available. In the nuclear staining package, the system can be trained to recognize nuclei with a minimum pixel intensity that corresponds to positive staining. By carefully fine-tuning of the shape and intensity settings for each individual immunohistochemical staining, we verified that the system only counted positively stained nuclei. For each TMA section, several random cores were evaluated by visual inspection after automatic analysis in order to verify that the system correctly identified positively stained nuclei. Automatic analysis of the percentage of positively stained nuclei (nuclear expression) was performed by the Ariol system for each individual tissue core.

### Statistical analyses

Data were analyzed in consultation with a statistician (H.P.) using SPSS 20.0 for Windows (SPSS Inc, Chicago, USA). The mean percentage of positive nuclei of the three tumor cores was calculated for each individual patient and this percentage was used for all statistical analyses. Normality of the data was tested using the Shapiro-Wilk test. Non-parametric Wilcoxon signed-rank tests were performed to assess the differences in mean nuclear expression between the paired tumor and normal tissues (n = 29) for each of the individual markers. The Cox proportional hazard model was used for univariate and multivariate survival analyses of individual and combined markers. Covariates included in all multivariate analyses were age at operation, gender, TNM tumor stage (tumor stages I-III), tumor location, tumor size, microsatellite stability (MSS) status. Additionally, covariates tumor in the follow up and adjuvant therapy were entered as time-dependent covariates. Patients in the study cohort (TNM stage I and II colon patients only) were divided into high and low expression groups based on the median expression of each of the markers separately. Based on the cellular function of each of the histone modifications, we expected low H3K4me3, high H3K9me3 and high H4K20me3 to be associated with a better prognosis (“all favorable”). For combinatorial analyses, patients were divided into groups based on the number of favorable markers (all favorable, 1 favorable, 2 favorable and all unfavorable). Univariate and multivariate trend analyses were performed using the group numbers as continuous variables to assess the influence of the combined markers on patient survival and tumor recurrence. Resulting hazard ratios (HR) represent the HR for each unit of increase (increase in group number, and hence an increase in the number of markers showing unfavorable expression). Overall survival (OS) was defined as the time from surgery until death (by any cause). Disease-specific survival (DSS) was defined as the time from surgery until death by colorectal cancer. Loco-regional recurrence-free survival (LRRFS) was defined as the time from surgery until the occurrence of a (loco)regional recurrence or death by cancer. Distant recurrence-free survival (DRFS) was defined as the time from surgery until the occurrence of a distant recurrence or death by cancer. Cumulative incidence curves were made for DSS, LRRFS and DRFS, accounting for competing risks [18]. Kaplan-Meier curves (for OS) or cumulative incidence curves (for DSS, LRRFS and DRFS) were used to visualize differences between the three patient groups for OS. For all statistical analyses, two-sided p-values ≤ 0.05 were considered as statistically significant, and p-values 0.05 < p ≤ 0.1 were considered a trend.

## Results

### Patient selection for statistical analyses

In this study, we analyzed 254 patients with TNM stage I-III colorectal cancer, with no prior history of cancer or preoperative treatment and of whom complete clinicopathological data were available (Table 1). Combined marker analyses, based on the number of favorable markers, showed statistically significant discrimination between patient groups in early-stage (TNM stage I and II) colon cancer (n = 121). Multivariate trend analyses showed significant differences between the patient groups for patients with TNM stage I or II colon cancer (p = 0.005), but no significant differences for patients with TNM stage I or II rectal cancer (p = 0.256). For patients with TNM stage III, no significant differences were observed for patients with either colon (p = 0.7) or rectal cancer (p = 0.6). Together, these results indicate prognostic value of H3K4me3, H3K9me3 or H4K20me3 expression in early-stage colon cancer patients. Therefore, patients with TNM stage III colorectal cancer or TNM stage I and II rectum cancer were excluded from further analyses. The resulting patient cohort consisted of 121 patients with TNM stage I or II colon cancer, with a mean follow-up of 9.4 years.

**Table 1:** Patient characteristics of the study cohort

	**Study cohort**		***Colon stage I + II***	
	***(n = 254)***		***(n = 121)***	
	**N**	**(%)**	**n**	**(%)**
Age at randomization				
<50	32	12.6	11	9.1
50-75	161	63.4	80	66.1
>75	61	24	30	24.8
Gender				
Male	128	50.4	61	50.4
Female	126	49.6	60	49.6
TNM stage				
I	53	20.9	30	24.8
II	113	44.5	91	75.2
III	88	34.6		
Tumor location				
Colon	187	73.6	121	100
Rectum	67	26.4		
Tumor size				
Mean	4.69		4.57	
Standard error	2.32		0.21	
MSS status				
MSS	175	68.9	20	16.5
MSI	34	13.4	75	62
Unknown	45	17.7	26	21.5
Tumor in follow up				
No	215	84.6	105	86.8
Yes	39	15.4	16	13.2
Adjuvant therapy				
No	206	81.1	117	96.7
Yes	48	18.9	4	3.3

Patient characteristics are shown for both the study cohort (n = 254) and the patients with TNM stage I and II colon cancer as used for the statistical analyses (n = 121). Patient selection was based on availability of FFPE tissues, available data for all three markers, and information about the listed covariables. The study cohort selection was representative for the entire colorectal cancer series.

### Nuclear expression in tumor versus normal tissues

**Figure 1:**
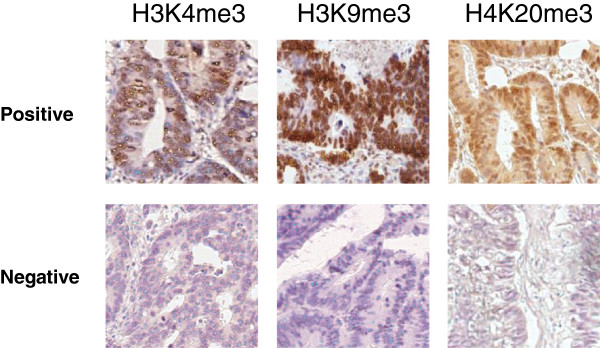
**Correct identification of positively stained and negative nuclei for each individual marker.** The Ariol system trainer overlay shows correct identification of positive (indicated by yellow dots) and negative (blue dots) nuclei in tumor tissues. TMA slides were scanned using a 20x magnification. Shown for all individual markers are positively stained nuclei (*top row*) and negative tumor cores (*bottom row*).

**Figure 2:**
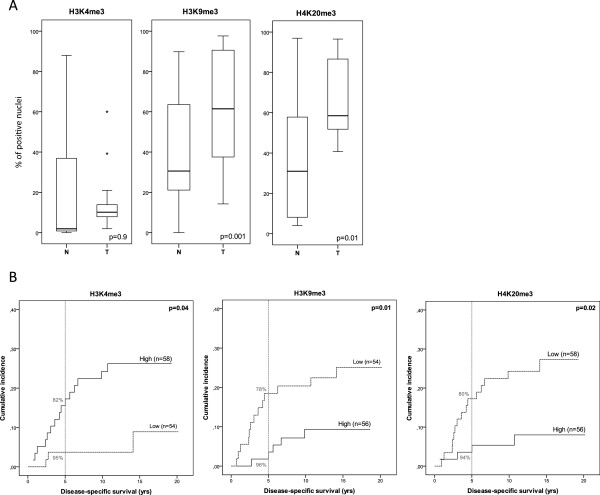
**Nuclear expression of individual markers. (A)**. Displayed are differences in nuclear expression, measured as the percentage of positively stained nuclei (y-axis), between normal and tumor tissues (n = 29). Boxplots show the median and range of expression of each of the individual markers in normal (N) and tumor (T) samples (x-axis). P-values represent statistical differences between normal and tumor samples, calculated using the Wilcoxon signed rank test. **(B)**. Cumulative incidence curves, accounting for competing risks, showing the difference in survival between high and low expression groups of each of the individual markers. 5-year survival rates are included as percentages (in gray); p-values represent the statistical differences between the two patient groups in multivariate analyses. Numbers of patients in each group are indicated in each figure (n).

Comparison of expression between paired tumor and normal tissues was preceded by testing normality of expression distribution data in the stage I and II colon cancer tissues per histone modification using the Shapiro-Wilk test. As the data of the individual markers were not normally distributed, we used the Wilcoxon signed-rank test to compare the expression in the paired tumor and normal tissues (n = 29). Marker expression was defined as the percentage of positively stained nuclei per tissue core. Representative staining of tumor tissue cores is shown in Figure 1. Statistically significant differences between tumor and normal samples were observed for H3K9me3 (p = 0.001) and H4K20me3 (p = 0.01), but not for H3K4me3 (p = 0.9) (Figure 2A).

### Survival analyses of individual markers

Median expression of each individual marker was used to divide the patients into high and low expression groups. The median expression for each of the individual markers in tumor tissues was 12.1% for H3K4me3, 65.5% for H3K9me3 and 65.4% for H4K20me3. Low expression of H3K4me3 was associated with better patient survival and lower chances of tumor recurrence (Figure 2B). In contrast, high expression of both H3K9me3 and H4K20me3 was associated with better patient survival and lower chances of tumor recurrence in our study cohort (Figure 2B). These findings are also reflected in the 5-year survival rates (Table 2). Both univariate and multivariate Cox regression analyses show significant differences between the low and high expression patient groups with respect to DSS, LRRFS and DRFS (Table 2).

**Table 2:** Survival analyses single markers in TNM stage I and II colon cancer patients

		**OS**	**DSS**	**LRRFS**	**DRFS**
**H3K4me3**					
*Univariate*	p-value	0.4	**0.02**	**0.01**	**0.01**
	HR	1.26	4.45	3.54	3.63
	(95% CI)	(0.75-2.11)	(1.29-15.38)	(1.32-9.49)	(1.36-9.73)
*Multivariate*	p-value	0.3	**0.04**	**0.01**	**0.01**
	HR	1.36	3.79	3.86	3.57
	(95% CI)	(0.79-2.33)	(1.06-13.56)	(1.38-10.77)	(1.29-9.81)
*5-year survival rates*	Low expression	73%	95%	93%	94%
	High expression	76%	82%	74%	77%
**H3K9me3**					
*Univariate*	p-value	*0.07*	**0.02**	*0.07*	**0.02**
	HR	0.61	0.30	0.47	0.36
	(95% CI)	(0.36-1.04)	(0.12-0.86)	(0.21-1.08)	(0.16-0.85)
*Multivariate*	p-value	0.2	**0.01**	**0.05**	**0.01**
	HR	0.69	0.26	0.42	0.29
	(95% CI)	(0.39-1.24)	(0.09-0.77)	(0.17-1.01)	(0.12-0.75)
*5-year survival rates*	Low expression	64%	78%	71%	74%
	High expression	86%	96%	92%	92%
**H4K20me3**					
*Univariate*	p-value	0.1	**0.01**	**0.02**	**0.04**
	HR	0.67	0.24	0.34	0.41
	(95% CI)	(0.40-1.12)	(0.08-0.72)	(0.14-0.81)	(0.18-0.95)
*Multivariate*	p-value	**0.02**	**0.02**	**0.008**	**0.01**
	HR	0.51	0.21	0.29	0.31
	(95% CI)	(0.29-0.89)	(0.06-0.67)	(0.12-0.72)	(0.13-0.77)
*5-year survival rates*	Low expression	67%	80%	74%	77%
	High expression	80%	94%	90%	91%

Shown are the results of the univariate and multivariate analyses of all individual markers in TNM stage I and II colon cancer patients, with all p-values and hazard ratios (HR) with their 95% confidence intervals (95% CI). OS = overall survival, DSS = disease-specific survival, LRRFS = locoregional recurrence free survival, DRFS = distant recurrence free survival. The low expression group (below-median expression) was used as reference group. 5-year survival rates are given for both low and high expression groups. Statistically significant differences (defined as p < 0.05) are shown in **bold**, trends (p < 0.1) in *Italic*.

### Survival analyses of combinations of two markers

We analyzed the prognostic value of combinations of two of the histone methylation markers. As both H3K4me3 and H3K9me3 are mostly found on gene promoter regions, and H4K20me3 and H3K9me3 in constitutive chromatin at pericentric regions, we hypothesized that these combinations of two histone modifications would result in better stratification of patients as compared to the individual markers. Multivariate analyses showed that combining the histone modifications indeed resulted in better separation of the patient groups with respect to patient survival and tumor recurrence. For the combination of gene promoter-associated modifications H3K4me3 and H3K9me3, we observed that the patient group with the most unfavorable expression pattern (high H3K4me3 and low H3K9me3) showed the shortest disease-free survival (trend analysis HR 2.05; p = 0.004) and distant recurrence-free survival (trend analysis HR 1.96; p = 0.001) as compared to the other patient groups. For the combination of pericentric region-associated modifications H4K20me3 and H3K9me3, the group with the most favorable expression (high expression of both markers) showed significantly better disease-free survival (HR 2.01; p = 0.005) and distant recurrence-free survival (HR 1.77; p = 0.004) as compared to the other patient groups.

### Survival analyses of H3K4me3, H3K9me3 and H4K20me3 combined

**Figure 3:**
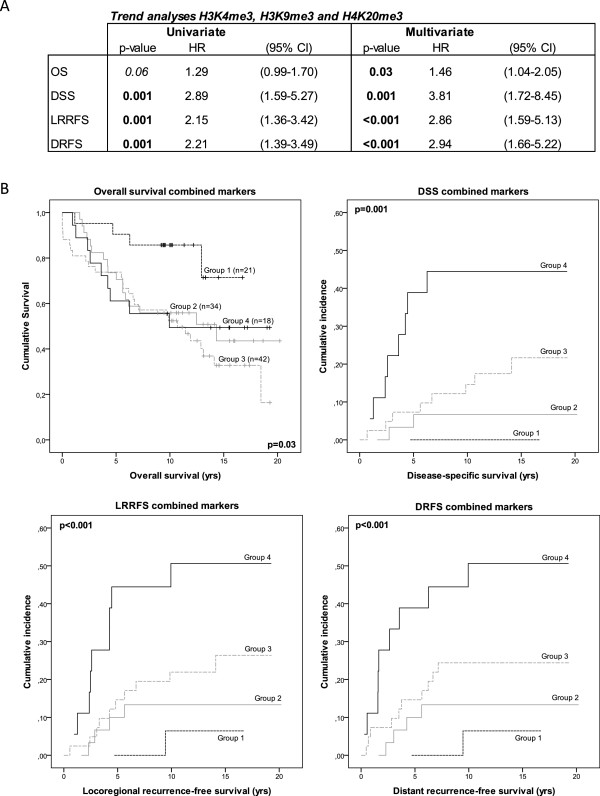
**Univariate and multivariate trend analyses of all markers combined. (A)**. Results of the univariate and multivariate trend analyses of combined markers H3K4me3, H3K9me3, and H4K20me3. HR represents the hazard ratio for each unit of increase, thus each additional marker showing unfavorable expression. 95% CI: 95% confidence interval for each HR. OS: overall survival; DSS: disease-specific survival; LRRFS: locoregional recurrence-free survival; DRFS: distant recurrence-free survival. **(B)**. Kaplan-Meier curves are shown for OS, including the number of patients in each patient group (n), based on the number of markers showing unfavorable expression. Patients were divided into the following groups: all favorable (group 1; H3K4me3 low and both H3K9me3 and H4K20me3 high), one out of three unfavorable (group 2), two out of three unfavorable (group 3), and all unfavorable (group 4; H3K4me3 high and both H3K9me3 and H4K20me3 low). Cumulative incidence curves, accounting for competing risks, are shown for DSS, LRRFS and DRFS. Multivariate p-values have been included in each of the combined marker graphs.

To further improve the stratification of patients, we performed Cox regression survival analyses using the combined expression patterns of all three markers H3K4me3, H3K9me3 and H4K20me3. Low expression of activating modification H3K4me3 and high expression of silencing modifications H3K9me3 and H4K20me3 was expected to be associated with good prognosis, and was therefore used as the “all favorable” reference group. Patients were divided into 4 groups, based on the number of markers showing clinically favorable or unfavorable expression. This resulted in the following grouping: all favorable (group 1; H3K4me3 low and both H3K9me3 and H4K20me3 high), one out of three unfavorable (group 2), two out of three unfavorable (group 3), and all unfavorable (group 4; H3K4me3 high and both H3K9me3 and H4K20me3 low). Both univariate and multivariate trend analyses showed that the more markers showed unfavorable expression, the shorter the patient survival (DSS) and recurrence-free survival times (both LRRFS and DRFS) (Figure 3A). The survival plots of OS, DSS, LRRFS and DRFS are shown in Figure 3B. Hazard ratios for the individual patient groups could not be calculated accurately, as not enough events (either death or recurrence of the tumor) occurred in the reference group (group 1; Figure 3B). Therefore, using multivariate trend analyses, we calculated hazard ratios for each additional marker showing unfavorable expression, as compared to the “all favorable” reference group. The calculated HRs were 1.46 (1.04-2.05; p = 0.03) for OS, for DSS 3.81 (1.72-8.45; p = 0.001) for DSS, 2.86 (1.59-5.13; p < 0.001) for LRRFS and 2.94 (1.66-5.22; p < 0.001) for DRFS. Combining all three markers resulted in better stratification and separation of the patient groups as compared to the single markers or the combinations of only two of the studied markers.

## Discussion

Aberrant gene expression is a common feature of cancer cells, which is caused by a combination of gene mutations and aberrant regulation of gene expression by epigenetic mechanisms, including DNA methylation, microRNAs and histone modifications. Histone modifications play a crucial role in many cellular processes during embryonic development, cell proliferation and cellular differentiation [2]. In cancer, aberrant expression of histone modifications has been described frequently [1]. Therefore, we investigated the nuclear expression of three well-studied histone modifications in colon cancer.

In this study, we found that nuclear expression of histone trimethylation on H3K4, H3K9 and H4K20 has prognostic value in early-stage colon cancer. Changes in expression of key histone modifications are found in early-stage tumors, which would be expected because tumor cells require instant changes in gene expression and chromatin structure in order to promote cell proliferation and tumor cell survival. Several epigenetic factors have been shown to be altered in early-stage cancer, including histone-modifying enzymes and histone modifications [19,20], DNA methylation [21,22] and microRNAs [23]. We only observed differences between the patient groups in colon tumors, whereas in rectum tumors no difference was observed. The observed differences between the colon and rectum tumors with respect to the studied histone modifications may be due to differences in biology of the tissues of origin. Several other studies have suggested that rectum and colon tumors show differential gene expression signatures [24,25]. This could be due to changes in epigenetic regulatory mechanisms. Detection of aberrant expression of prognostic histone modifications, such as described in this study in early-stage colon cancer, could facilitate the risk assessment and subsequent decisions for treatment for specific patient groups at early stages of the disease.

The results of the survival analyses of the individual markers reflect our expected results based on the cellular functions of the respective histone modifications. Trimethylation of H3K4 is a modification found on gene promoter regions and is associated with activation of gene transcription [26], and higher expression of H3K4me3 in tumors could lead to aberrant gene transcription, including genes required for cell survival, proliferation and migration. In literature, poor prognosis was indeed reported for patients with tumors showing high expression of H3K4me3 [4]. Histone modification H4K20me3 is a known repressive mark [13], and key modification regulating compaction of the chromatin in pericentric regions, which makes it crucial for proper chromosome segregation during cell division and for maintenance of genome integrity [12]. Consequently, loss of H4K20me3 was expected to be associated with a worse prognosis for the patient, which has indeed been shown in literature [9]. Finally, for H3K9me3, literature shows conflicting results with respect to patient survival and prognosis [6-8,27], depending on the type of cancer. Histone modification H3K9me3 is associated with silencing of gene transcription [26], and can hence be involved in aberrant silencing of tumor suppressor genes (i.e. DCC [28]). On the other hand, H3K9me3 prevents aberrant expression of (onco)genes and represses the abundant repetitive sequences in the genome [29-31]. On the basis of the function of H3K9me3 as a silencing modification, we expected H3K9me3 expression to be comparable to H4K20me3 expression with respect to clinical outcome. Our results confirmed the hypotheses based on these individual functions, as high expression of H3K4me3 and low expression of H3K9me3 and H4K20me3 are correlated with shorter patient survival and higher chances of tumor recurrence.

Combined marker analyses showed that favorable expression of all markers (low H3K4me3, high H3K9me3 and high H4K20me3, as based on the individual marker analyses) was associated with the best prognosis with respect to patient survival and tumor recurrence. With each additional marker showing more unfavorable expression, the HR increased significantly about 3-fold for DSS, LRRFS and DRFS, indicating that combining all three methylation marks resulted in better separation of the patient groups as compared to individual markers. Combining multiple markers in survival analyses can thus be beneficial in identifying high-risk patient groups and to determine treatment strategies accordingly. To our knowledge, this is the first study to combine these three markers in survival analyses. In literature, multiple histone modifications have been studied in cancer tissues but have never been combined in survival analyses [8,32-35]. In addition, expression of histone modifications was not always correlated to clinical outcome [36], or were found to have no prognostic value in cancer [37].

## Conclusions

In conclusion, in this study we have shown that combined nuclear expression of histone trimethylation on H3K4, H3K9 and H4K20 is prognostic in early-stage colon cancer and that combined expression of the three histone modifications provides better stratification of patient groups and therefore provides a better risk assessment as compared to the individual markers. The clinically prognostic value of the histone modifications presented in this study underlines the consequences of epigenetic dysregulation in tumorigenesis.

## Abbreviations

TNM: Tumor, nodes, metastasis; H3K4me3: Trimethylation of lysine 4 on histone H3; H3K9me3: Trimethylation of lysine 9 on histone H3; H4K20me3: Trimethylation of lysine 20 on histone H4; HR: Hazard ratio; IHC: Immunohistochemistry; OS: Overall survival; DSS: Disease-specific survival; LRRFS: Locoregional recurrence-free survival; DRFS: Distant recurrence-free survival.

## Competing interests

None of the authors have any conflict of interest to declare.

## Authors’ contributions

AB, IGB, AvH, CvdV and PK have been involved in conception and design of the study. AB, IGB, AvH, WdG, HH and EZ have been involved in acquisition of the data. AB, IGB and HP have been involved in analysis and interpretation of the data. AB, IGB, CvdV and PK have been involved in drafting the manuscript. All authors have critically revised the manuscript and have approved the final version for submission.

## Pre-publication history

The pre-publication history for this paper can be accessed here:

http://www.biomedcentral.com/1471-2407/14/531/prepub
